# Determination of very long-chain polyunsaturated fatty acids from 24 to 44 carbons in eye, brain and gonads of wild and cultured gilthead sea bream (*Sparus aurata*)

**DOI:** 10.1038/s41598-022-14361-0

**Published:** 2022-06-16

**Authors:** Roque Serrano, Juan C. Navarro, Carlos Sales, Tania Portolés, Óscar Monroig, Joaquin Beltran, Félix Hernández

**Affiliations:** 1 Research Institute for Pesticides and Water (IUPA), University Jaume I, Av. Sos Baynat S/N, 12071 Castellón, Spain; 2 Research Unit of Marine Ecotoxicology, UJI, Associated Unit to CSIC by IATS, Av. Sos Baynat S/N, 12071 Castellón, Spain; 3 Institute of Aquaculture Torre de la Sal (IATS), CSIC, 12595 Ribera de Cabanes, S/NCastellón, Cabanes Spain

**Keywords:** Biochemistry, Chemistry

## Abstract

Very long-chain (> C24) polyunsaturated fatty acids (VLC-PUFA) play an important role in the development of nervous system, retinal function and reproductive processes in vertebrates. Their presence in very small amounts in specific lipid classes, the lack of reference standards and their late elution in chromatographic analyses render their identification and, most important, their quantification, still a challenge. Consequently, a sensitive and feasible analytical methodology is needed. In this work, we have studied the effect of chain length, as well as the number and position of unsaturations (or double bonds) on the response of GC-APCI-(Q)TOF MS, to establish an analytical method for VLC-PUFA quantification. The developed methodology allows the quantification of these compounds down to 2.5 × 10^–3^ pmol/mg lipid. The reduction of VLC-PUFA levels in lipid fractions of the organs from the herein sampled farmed fish suggesting a yet undetected effect on these compounds of high vegetable oil aquafeed formulations, that currently dominate the market.

## Introduction

Long-chain (C_20_–C_24_) polyunsaturated fatty acids (LC-PUFA) are regarded as very important compounds with physiologically critical functions in vertebrates including humans^1^. Among them, the n-3 (or “omega-3”) eicosapentaenoic acid (EPA, 20:5n3) and docosahexaenoic acid (DHA, 22:6n-3), and the n-6 arachidonic acid (ARA, 20:4n-6), are important for normal growth and development, and, in mammals, they play pivotal roles in the inflammatory response. Importantly, dietary consumption of EPA and DHA has been found beneficial in cardiovascular and cerebrovascular conditions, and some types of cancer^2,3^. Marine fish, particularly oily species, represent a major source of n-3 LC-PUFA in the humans diet, and as a consequence, these compounds have been of special concern for researchers^4^. Farmed fish represent an ever-increasing portion of fish consumed by humans^5^. The rapid expansion of aquaculture in the last decades has urged the need to use non-fish derived raw materials in the feed. Consequently, aquafeed formulations have remarkably decreased the levels of LC-PUFA, hence impacting the nutritional value of the final product because of the reduced levels of the health-promoting n-3 LC-PUFA such as EPA and DHA, and interestingly, compromising the health of the farmed fish itself whereby LC-PUFA have critically important functions as described above for mammals.

LC-PUFA are biosynthetic precursors of the so-called very long-chain (> C_24_) PUFA (VLC-PUFA) and, therefore, the abovementioned reduction of LC-PUFA in fish diets has been hypothesized to compromise the physiological demands of VLC-PUFA in farmed fish. It is now well established that the elongation of very long-chain fatty acid protein 4 (Elovl4) is a pivotal enzyme determining the bioconversion of LC-PUFA into VLC-PUFA^6,7^. VLC-PUFA have been found in low concentrations in retina, sperm and brain of mammals, as a part of phosphatidylcholine (PC), sphingomyelin (SM) and cerebrosides (CE)^6,7^, and have been therefore considered essential for development of nervous system, retinal function and reproductive processes^8–10^. Since their detection in bovine retinas by Aveldaño et al*.* in 1987^11^, the study of VLC-PUFA has been mainly focused on mammals^8,12–14^. Surprisingly, the detection and characterization of VLC-PUFA has been barely studied in fish despite the potential detrimental effects noted above associated with a reduction of metabolic precursors (i.e. LC-PUFA) in current aquafeed formulations^15^.

VLC-PUFA are present in very small amounts in specific lipid classes of particular tissues including brain, retina and gonads. Moreover, the absence of commercially available standards and their late elution in standard chromatographic analyses has hindered the identification of VLC-PUFA in animals^6^. Liquid chromatography (LC) coupled to different mass spectrometers (MS) (with electrospray ionization (ESI) or atmospheric pressure chemical ionization (APCI)) in normal and reverse phase has been applied to the analysis of lipids containing VLC-PUFA^6,12,16–18^. However, although a derivatization step to fatty acid methyl esters (FAME) is required for fatty acid determination, gas chromatography (GC) coupled to MS with electron ionization (EI) is the most frequently used technique for the analysis of VLC-PUFA^19,20^. Pioneer studies from the 80’s reported VLC-PUFA of up to 40 carbons^21^. Nevertheless, resolution of quadrupole analyzers (over 1 Da), and deficient chromatographic separation, made very challenging to obtain accurate and reliable identification of VLC-PUFA. Moreover, the use of EI source provokes an extensive fragmentation of FAMEs and involves the partial or total loss of the highly diagnostic molecular ion (M^+·^) generating the same EI spectra for unsaturated FAMEs (both n-6 and n-3) with different chain length, which hinders the identification process.

Recent studies have applied APCI source with quadrupole time-of-flight mass analyzers (QTOF) coupled to GC to the qualitative analysis of VLC-PUFA in fish^22^. This soft ionization technique has allowed to obtain the parent molecule, enabling the accurate identification of VLC-PUFA by means of measuring protonated molecule at accurate mass. Moreover, the study of the mass spectrometric behavior enables the discrimination between n-3 and n-6 compounds^22^. The application of this technique has also allowed, for the first time, the identification of VLC-PUFA with chains up to 44 carbons in the SM and PC fractions in the total lipids from fish based on their accurate mass and fragmentation pattern^23^.

While the above-mentioned absence of reference standards makes difficult the identification of VLC-PUFA, their quantitation, aggravated by their extremely low abundance pointed out above, is almost an unexplored field. Furland et al*.*^24^ expressed semi-quantitative results of VLC-PUFA content in rat testis as percentages of the total fatty acids in each lipid class. Méndez et al*.*^25^ and Liu et al*.*^13^ based the quantitative analysis of VLC-PUFA on the internal standard (IS) method by the determination of mass response factor, extrapolating the data from existing standards C30:0-C36:0. In the above studies, the extrapolation of the response factor did not take into account the dependence of the instrument response on chain length, nor the degree and the position of unsaturations. Liu et al*.*^14^ developed a method to quantify VLC-PUFA expressed as fatty acids molar percentage, but the response of a single quadrupole mass analyzer shows trends caused by chain length, number of unsaturations and their position, and the need of performing extrapolations.

The methodology established in this work is able to identify, and also quantify, VLC-PUFAS with chains up to 44 carbons in fish organs, improving previous methodologies taking advantage of the GC-APCI-QTOF, not used before to the best of our knowledge. The effect of chain length, number of unsaturations and their position to the response of GC-APCI-QTOF was studied in order to establish a quantification method of VLC-PUFA, taking into account the possible variations of the VLC-PUFA response respect PUFA standards available. This methodology was applied in the identification and quantification of VLC-PUFA known to be present in the PC, SM and CE lipid fractions of eye, brain and gonads of wild and culture gilthead sea bream (*Sparus aurata*)^23^, providing valuable information, for future studies in the fields of lipid biochemistry and aquaculture, about the presence of these novel molecules in wild and farmed fish tissues.

## Results and discussion

### Identification of VLC-PUFA

Like in a previous work^23^, the identification of VLC-PUFA was conducted looking for a chromatographic peak for each of the corresponding [M+H]^+^ ions using 0.02 Da narrow window eXtracted Ion Chromatograms (nw-XICs). Mass spectra of the different VLC-PUFA peaks were studied for further identification of the compounds. The mass accuracy of [M+H]^+^ was evaluated in the low-energy (LE) function, and mass errors were below 3.2 ppm in all cases. Table S1 shows the mass spectrometry parameters used for the identification of targeted compounds (for more details see Serrano et al*.*^23^).

### Quantification of VLC-PUFA

**Figure 1:**
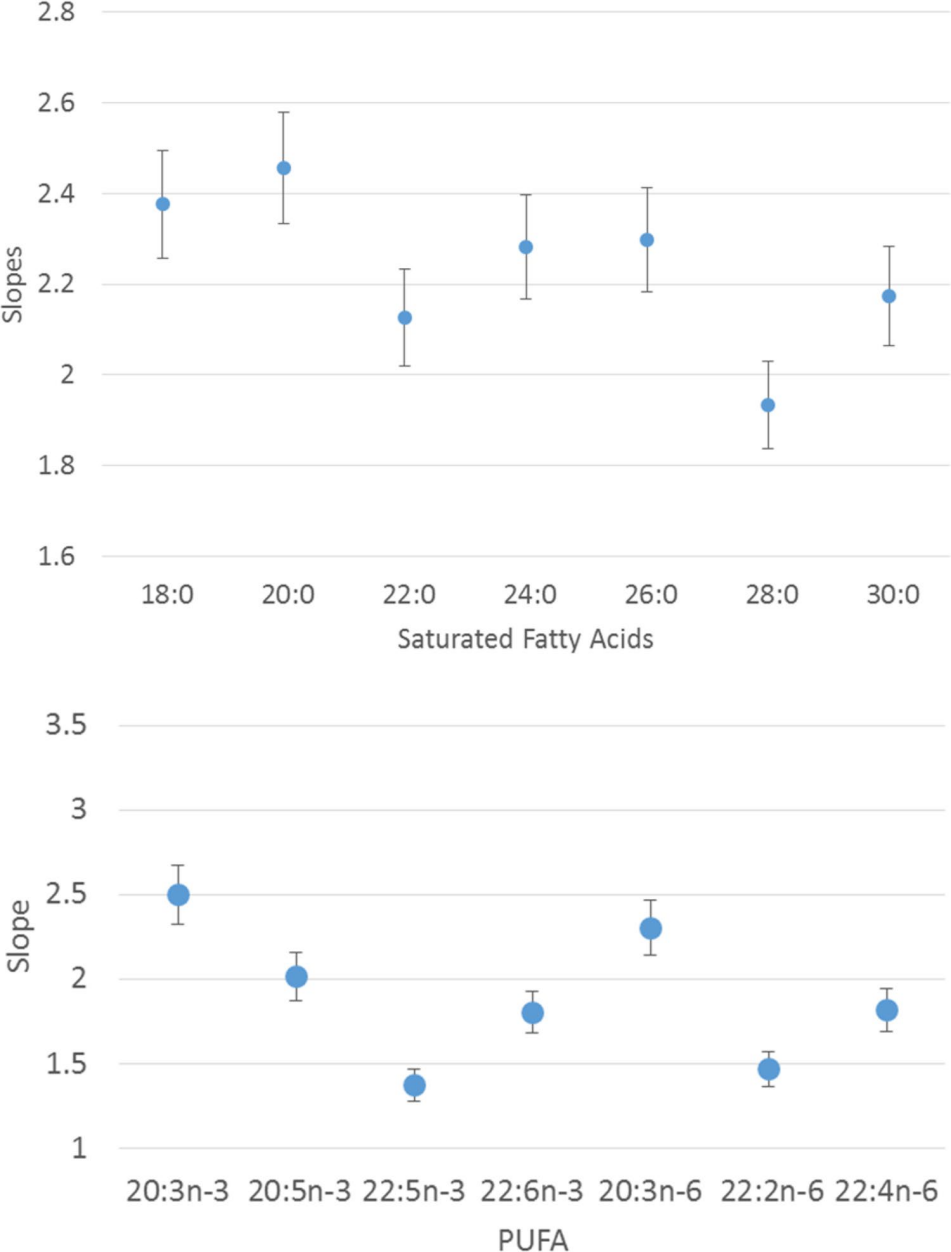
Slopes of the calibration curves from the responses of saturated fatty acids and PUFA methylated reference standards available. Error bars: standard deviation (n = 6).

In order to develop a quantification method based on Q-TOF mass analyzer with APCI source, slopes of calibration curves (n = 6) from equimolar mixtures (0.002, 0.02, 0.2, 2 and 4 pmol/µL) of each saturated FAME (18:0, 20:0, 22:0, 24:0, 26:0, 28:0 and 30:0) and PUFA methyl esters (20:3n-3, 20:3n-6, 20:4n-3, 20:4n-6, 20:5n-3, 22:2n-6, 22:4n-6 and 22:6n-3) were studied to elucidate the effect of chain length, as well as position and number of unsaturations, on the instrumental response. Comparison of slope values from saturated FAMEs and PUFA calibration curves, respectively, using Student’s t-test based in both the standard error of the regressions and the standard error of the slopes^26^ revealed no statistical differences and no trends regarding length of chains and position and number of unsaturations (Fig. 1).

Slopes from FAMEs presented a variation coefficient of 5.6% with a confidence interval at 95% of ± 4.9% (n = 7). Regarding PUFA methyl esters, statistical analysis showed also that slopes were statistically equal and position and number of unsaturations did not show relation with instrumental response. Values of coefficient of variation and confidence interval at 95% (n = 8) were 4.6% and ± 4.2%, respectively (n = 8) (Figs. S1, S2). In view of the results, the points of the calibration curves for VLC-PUFA quantification were calculated as the arithmetic mean of the responses of each of the eight available PUFA standards (n = 6). Hence, the response obtained by GC-APCI-(Q)TOF MS did not show appreciable trends related to the characteristics of the molecules, minimizing possible biases in the determination of very long chain molecules.

Table 1 shows the uncertainties for each point of calibration curve calculated as the arithmetic mean from eight calibration curves corresponding to each PUFA methyl ester reference standard available (see Fig. S1), with different number of carbons, number of unsaturations and unsaturation positions. The precision of the calibration curves obtained from the selected PUFA standards and the lack of trends in the response of QTOF due to long chain or unsaturations, confirmed the possibility of the quantitation of VLC-PUFA with acyl chains longer than 24 carbons, avoiding as far as possible biases due to the differences among molecule structures. The limits of detection (LOD) were estimated down to 2.5 × 10^–4^ pmol/mg Lipid (limits of quantification down to 2.5 × 10^–3^ pmol/mg Lipid).

**Table 1:** Uncertainties of the experimental calibration curve calculated as the arithmetic mean from eight calibration curves corresponding to PUFA methyl ester standards.

Concentration of calibration point (pmol/µL)	Uncertainty (%)*	Uncertainty (pmol/µL)
0.002	5.5	0.0002
0.02	4.9	0.001
0.2	4.3	0.002
2	2.8	0.006
4	4.05	0.08

*Calculated at 95% confidence level.

Table 2 shows recoveries obtained in the validation experiments. In all cases, the recovery of the standards assayed was satisfactory.

**Table 2:** Validation data of the proposed method at two levels of concentration for standards and internal standard (n = 5).

Standard	Retention time (min)	Concentration (pm/µL)	Recovery ± rsd (%)
C28:0	8.98	0.02	124 ± 7
		2	112 ± 8
C30:0	9.58	0.02	71 ± 9
		2	102 ± 7
C27:0 (IS)	8.67	0.25	99 ± 10

*rsd *relative standard deviation*.*

**Table 3:** Concentrations of VLC-PUFA (pmol/mg Lipid) found in the eyes, brain and gonads samples from wild (W) and farmed (F) specimens of gilthead sea bream. (RSD (%) range: 6–40) (more details in Supplementary information).

Compounds		Eyes			Brain			Gon		
		SM	PC	CE	SM	PC	CE	SM	PC	CE
24:4n-6	W	0.028	0.030	0.011	0.028	0.030	0.011	0.024	0.25	0.017
	F	0.005	0.05	0.005	0.005	0.05	0.005	< LOQ	nd	na
24:5n-6	W	< LOQ	0.044	0.004	0.036	0.229	0.014	0.044	0.214	0.036
	F	nd	< LOQ	na	0.005	0.363	0.005	< LOQ	0.186	na
24:6n-3	W	0.010	0.143	0.006	0.083	0.372	0.022	0.174	0.475	0.030
	F	nd	0.033	na	0.005	0.937	0.005	< LOQ	0.544	na
26:4n-6	W	0.005	0.014	< LOQ	0.004	0.021	0.006	0.009	0.052	0.00
	F	nd	nd	na	< LOQ	0.005	nd	nd	nd	na
26:5n-6	W	0.006	0.032	< LOQ	0.014	0.066	0.009	0.013	0.057	0.010
	F	nd	nd	na	0.005	0.012	< LOQ	< LOQ	0.050	na
26:6n-3	W	< LOQ	0.034	< LOQ	0.066	0.046	0.008	0.011	0.030	nd
	F	nd	nd	na	0.005	0.005	< LOQ	< LOQ	0.014	na
28:4n-6	W	0.008	0.014	< LOQ	0.006	0.010	0.002	0.007	0.022	nd
	F	nd	nd	na	< LOQ	0.005	nd	< LOQ	nd	na
28:5n-3	W	< LOQ	< LOQ	< LOQ	0.006	0.010	0.009	0.007	0.012	0.011
	F	nd	nd	na	0.005	0.005	< LOQ	< LOQ	nd	na
28:6n-3	W	< LOQ	0.042	< LOQ	0.007	0.029	0.004	0.008	0.018	nd
	F	nd	nd	na	0.005	0.005	nd	nd	< LOQ	na
30:4n-6	W	0.020	0.040	< LOQ	0.006	0.009	nd	0.007	0.021	nd
	F	nd	nd	na	< LOQ	0.004	nd	< LOQ	nd	na
30:5n-3	W	0.040	0.114	0.005	0.007	0.016	nd	0.010	0.042	nd
	F	nd	nd	na	0.005	0.005	nd	< LOQ	nd	na
30:6n-3	W	0.007	0.261	0.05	0.008	0.022	nd	0.013	0.023	nd
	F	nd	0.052	na	0.004	0.005	< LOQ	< LOQ	nd	na
32:4n-6	W	0.006	0.046	< LOQ	< LOQ	0.006	nd	0.007	0.019	nd
	F	nd	nd	na	nd	nd	nd	nd	nd	na
32:5n-3	W	0.016	0.306	0.006	0.006	0.012	nd	0.012	0.028	nd
	F	nd	0.021	na	0.004	0.005	nd	< LOQ	nd	na
32:6n-3	W	0.100	0.500	0.071	0.026	0.033	0.006	0.033	0.059	0.01
	F	0.015	0.516	na	0.005	0.005	< LOQ	< LOQ	nd	2Na
34:4n-6	W	0.004	0.006	nd	< LOQ	0.009	nd	0.007	0.018	nd
	F	nd	nd	na	< LOQ	< LOQ	nd	nd	nd	na
34:5n-3	W	< LOQ	0.012	< LOQ	0.006	0.013	nd	0.008	0.039	nd
	F	nd	nd	na	0.005	0.005	nd	< LOQ	< LOQ	na
34:6n-3	W	0.010	0.118	< LOQ	0.008	0.027	0.004	0.027	0.037	nd
	F	nd	0.019	na	0.005	0.005	nd	< LOQ	< LOQ	na
36:6n-3	W	0.006	0.017	< LOQ	0.0133	0.055	< LOQ	0.010	0.026	nd
	F	nd	nd	na	0.005	0.005	nd	< LOQ	< LOQ	na
38:6n-3	W	< LOQ	0.008	< LOQ	0.022	0.135	0.002	0.007	0.019	nd
	F	nd	nd	na	0.005	0.007	nd	< LOQ	< LOQ	na
40:6n-3	W	0.006	0.021	< LOQ	0.094	0.423	0.007	nd	0.016	nd
	F	nd	nd	na	0.005	0.153	< LOQ	na	< LOQ	na
42:6n-3	W	nd	< LOQ	nd	0.007	0.020	nd	nd	nd	nd
	F	nd	nd	na	< LOQ	< LOQ	nd	nd	nd	na
44:6n-3	W	nd	nd	nd	nd	0.004	nd	nd	nd	nd
	F	nd	nd	nd	nd	nd	nd	nd	nd	na

*SM* Sphingomyelin, *PC* Phosphatidylcholine, *CE* Cerebrosides, *nd* not detected,*na* not analyzed.

### VLC-PUFA in eye, brain and gonads from fish

**Figure 2:**
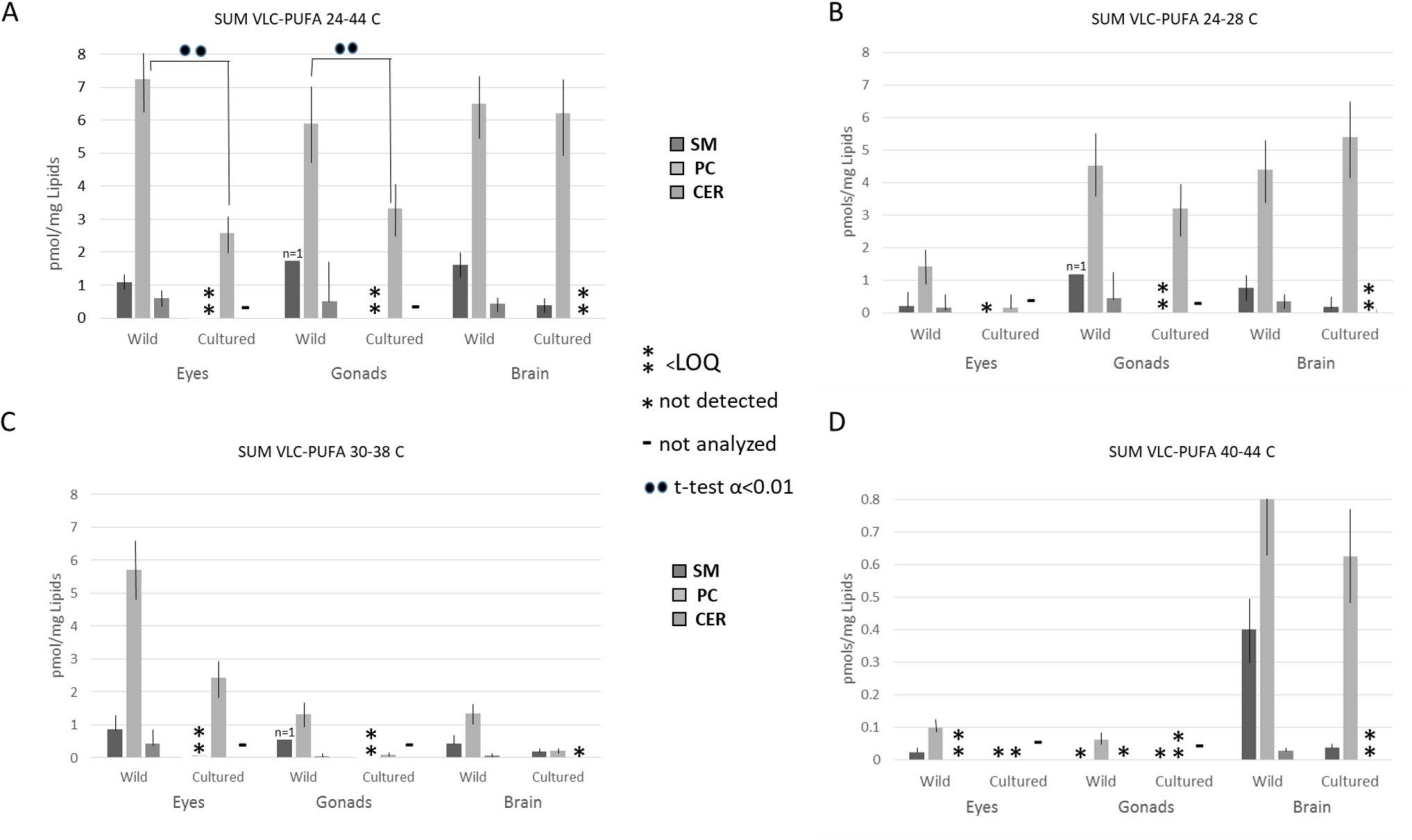
Concentrations (pmol/mg Lipid) of VLC-PUFA determined in lipid classes contained in eye, gonads and brain of wild and farmed specimens of gilthead sea bream. *SM* sphingomyelin, *PC* phosphatidylcholine, *CE* cerebrosides.

It is a well-known fact that the highest expression of *elovl4* genes is circumscribed to brain, eye and gonads of fish^1,27^. It has also been described that the expression patterns are linked to phenotypic influences, essentially the diet^28,29^. Table 3 shows the VLC-PUFA detected in selected lipid classes from gilthead sea bream organs. As it can be observed, molecules with chains up to 44 carbons were detected and, interestingly, these C_44_ VLC-PUFA were only present in PC from brain of wild *S. aurata* individuals. Figure 2A shows the concentration (pmol/mg Lipid) of total VLC-PUFA determined in lipid classes contained in the selected organs of wild and farmed specimens of gilthead sea bream (more details on VLC-PUFA concentrations in Tables S2 and S3). Quantitation shows that PC is the lipid class with the highest content of VLC-PUFA.

As can be observed in Fig. 2A, VLC-PUFA are especially abundant in the phosphatidylcholine fraction. Sphyngomiyelin and cerebrosides fractions present, in all organs analyzed, higher content of VLC-PUFA in wild specimens than in farmed, with levels close to LOQ. Nevertheless, in the phosphatidylcholine fraction, no differences between farmed and wild individuals were detected in brain, while eyes and gonads of farmed fish showed lower content of VLC-PUFA (*t*-test, 99% confidence level) than wild specimens. Considering that VLC-PUFA are biosynthesized in fish through Elovl enzyme-mediated reactions from LC-PUFA precursors^30^, these results suggest that diet can impact VLC-PUFA homeostasis and, potentially, the biological functions that these compounds exert in vision and reproduction. On one hand, natural diets of a carnivorous fish like the gilthead sea bream are rich in LC-PUFA and therefore, biosynthesis of VLC-PUFA guarantees the physiological demands in key tissues. On the other, compared to wild fish, dietary supply of LC-PUFA in farmed fish, albeit likely covering their minimum nutritional requirements, is largely lower due to the high inclusion levels of non-marine ingredients in current aquafeed formulations^31^. Thus provision of adequate levels of LC-PUFA in the diet affects more strongly eyes and gonads, being these organs more dependent on an adequate dietary provision of VLC-PUFA precursors (i.e. LC-PUFA)^1,32–36^ generally found in lower amounts in substituted diets^15,33,37^.

Figure 2B–D show the content of VLC-PUFA grouped in chain length segments, namely C_24_–C_28_ (short-chain VLC-PUFA), C_30_–C_38_ (medium-chain VLC-PUFA) and C_40_–C_44_ (long-chain VLC-PUFA). Short VLC-PUFA are more abundant in eyes, whereas the long-chain VLC-PUFA are mainly located in brain. Moreover, eyes preferentially concentrate medium-chain VLC-PUFA. To note, the amounts of VLC-PUFA with chains of less than 40 carbons detected in the different tissues are one order of magnitude lower than the rest and, interestingly, the SM of wild specimens seems particularly rich in these compounds, especially in brain, as compared to the cultured ones. Distribution by chain length may be relevant since it has been suggested that the properties of VLC-PUFA are directly linked to their structure presenting simultaneously properties of both saturated and polyunsaturated fatty acids in each of the extremes^6,38^.

In conclusion, the method developed in the present study allows the quantitation of VLC-PUFA in the lipids of target tissues of fish and reveals their distribution in different lipid classes. Furthermore, the application of the method to samples collected from wild and farmed individuals of the gilthead sea bream provides evidence of the impact of currently used feed formulations on VLC-PUFA biosynthesis. Although it can be argued that the present findings should be limited to the particular fish stocks/individuals sampled, the extensively documented phenotypic fingerprint on the fatty acid composition of cultured fish makes generalization tempting. Such generalization was beyond the objectives of the present work, but quantitation of VLC-PUFA will lay the groundwork for future studies linking their presence to gene expression and phenotypic performance to ascertain the effects of these compounds in visual acuity, fertility, breeding and growth^1,32,39,40^.

## Materials and methods

### Reagents and solvents

In this research, commercially available fatty acids and PUFA analytical standards were selected. Free form and methylated (ME) saturated fatty acids from 18:0 to 24:0 were purchased from Fluka (Zwijndrecht, The Netherlands), and from 26:0 to 30:0 from Dr. Ehrenstorfer (Augsburg, Germany). PUFA including 20:4n-3, 20:4n-6, 20:5n-3, 22:4n-3, 22:4n-6, 22:5n-3, 22:6n-3 and 24:5n-3 standards were purchased from Supelco (Bellefonte, PA, USA). Stock solutions (around 500 μg/mL, except 1 μg/mL for 24:0-ME and 26:0-ME) were prepared by dissolving solid reference standards in hexane or by diluting reference standard solutions in hexane and subsequently stored in a freezer at – 20 °C under a N_2_ atmosphere. Working solutions were prepared by diluting stock solutions in hexane.

Hexane (ultra-trace quality) was purchased from Scharlab (Barcelona, Spain). Chloroform (CHCl_3_), diethyl ether and toluene were purchased from Merck Millipore (Darmstadt, Germany). Methanol (MeOH) was purchased from VWR (Radnor, PA, USA). Sulfuric acid, glacial acetic acid and potassium chloride (KCl) were purchased from Panreac (Castellar del Vallés, Barcelona, Spain). Iodine and butylated hydroxytoluene (BHT, content > 99%), used as antioxidant, were purchased from Sigma Aldrich (St. Louis, MO, USA). All reagents were analytical grade. HPLC-grade water was obtained by purifying double distilled water in a Milli-Q Gradient A10 system (Millipore, Bedford, MA, USA) (for more details see Garlito et al*.*^22^).

### Instrumental

An Agilent 7890 N gas chromatograph (Palo Alto, CA, USA) equipped with an Agilent 7683 autosampler was coupled to a quadrupole orthogonal acceleration time-of-flight mass spectrometer, Xevo G2 QTOF (Waters Corporation, Manchester, UK), equipped with APGC v2.0 as ionization source, working in positive APCI mode. A fused silica VF-5HT capillary column with a length of 15 m × 0.32 mm i.d. and a film thickness of 0.10 μm (J&W Scientific, Folson, CA, USA) was used for GC separation. The oven temperature was programmed as follows: 130 °C (1 min); 20 °C/min to 340 °C; (2.5 min) with a total runtime of 14 min. Pulsed splitless (25 psi) injections of 1 μL were carried out at 280 °C with a splitless time of 1 min. Helium 99.999% (Praxair, Spain) was used as carrier gas at a flow of 4 mL/min.

The interface and ionization source temperatures were set to 340 °C and 150 °C, respectively. N_2_ was used as auxiliary gas at 200 L/h, as cone gas at 5 L/h and as make-up gas at 300 mL/min. The APCI corona discharge pin was operated at 1.7 μA and the cone voltage was set to 20 V.

The QTOF was operated at 2.5 spectra/s acquiring the mass range m/z 50–1000. The TOF MS resolution was approximately 15,000 (FWHM) at m/z 264. Acquisition was done in MSE mode in which two alternating acquisition functions with different collision energies were generated: the low-energy (LE) function, selecting a collision energy of 4 eV to avoid or minimize fragmentation, and the high-energy (HE) function, with a collision energy ramp ranging from 25 to 40 eV to obtain a greater range of fragment ions.

Heptacose (Sigma Aldrich, Madrid, Spain) was used for the daily mass calibration. Internal calibration was performed using octafluoronaphthalene (Sigma Aldrich, Madrid, Spain) as lock mass (monitoring the molecular ion, m/z 271.9872).

In order to work under proton transfer conditions, an uncapped vial containing water was placed in a designed holder into the APCI source door to enhance protonation. MS data were acquired in centroid mode and processed by the ChromaLynx XS application manager (within MassLynx v4.1; Waters). Mass-Fragment software (Waters) was used for mass spectra interpretation.

### Sampling and sample treatment

Eyes, brains and gonads (n > 5) were collected from wild (western Mediterranean) and farmed specimens of gilthead sea bream from local (Castellón, Spain) markets.

The study was reviewed and approved by the ethics committees of the Spanish Research Council (CSIC), Local Government (“Generalitat Valenciana”) and Institute of Aquaculture “Torre de la Sal” under the framework of project AGL 2013-40986-R (Spanish Government, Ministry of Economy and Competitiveness). All methods were performed in accordance with the relevant guidelines and regulations, including ARRIVE guidelines.

Adult specimens of gilthead sea bream were dissected and eyes, brains and gonads were collected and stored at − 20 °C until further analysis. Selection of organs and lipid classes was based on previous evidences of the presence of VLC-PUFA^22,23^. Crystalline lenses were removed from eyes. Total lipids were extracted using the method of Folch et al*.*^41^ Subsequently, an aliquot of total lipids (~ 200 mg) was further developed by thin layer chromatography (TLC 20 × 20 silica gel G60, Merck, Darmstadt, Germany) using a polar solvent system (methyl acetate:propan-2-ol:chloroform:methanol:0.25% (w/v) aqueous KCI (25:25:25:10:9 by vol). The three fractions where VLC-PUFA were detected in previous studies^22,23^, namely SM, PC and CE, were scrapped off the plate^42^, eluted in chloroform:methanol (2:1, v/v) containing BHT (0.01%, w/v), and used to prepare FAMEs^43^. FAME samples were stored in hexane containing BHT (0.01%) under nitrogen at -20ºC (for more details see Garlito et al.^22^ Prior to the analysis by GC-APCI-QTOF MS and GC-APCI-IMS-QTOF MS samples were dried with a gentle flow of N_2_ and reconstituted in 50 µL of hexane.

### Quantification of VLC-PUFA

A direct calibration with internal standard (IS 27:0) calculated as the arithmetic mean of the responses of each of the eight available PUFA standards was used to quantify VLC-PUFA. Calibration curves were made by triplicate with five concentrations (0.002, 0.02, 0.2, 2, 4 pmol/µL). Internal standard was added to standards and samples before injection. Goodness of the regressions were checked by means of residuals study. Trends were not observed and residuals values were below 5% in all cases. Values of r^2^ were higher than 0.99 in all cases. Homoscedasticity of the slopes were confirmed by means of F-test. The limit of detection (LOD) was estimated from the chromatograms of sample extracts fortified at the lowest level tested (i.e., 0.002 pmol/µL) for a signal-to-noise ratio of 3. LOQ was calculated as the concentration for a signal-to-noise ratio of 10.

### Validation

In order to verify quantitatively the whole procedure, samples of eyes were fortified with the saturated FA standards with greater number of carbons (28:0 and 30:0), and with the IS used (C27), with the aim of simulating the VLC-PUFA molecules as best as possible. Recovery experiments (n = 5) at two different levels for 28:0 and 30:0 (0.02 and 2 pmol/µL), and at 0.25 pmol/µL for 27:0 (concentration added of IS), were performed. The fortified samples were left to stand for 1 h prior to extraction. Precision, expressed as relative standard deviation (%) was calculated from five replicates processed in the same conditions.

### Data processing

Calculations and statistical tests were carried out using MS EXCEL 2013 (data analysis module). Slopes were statistically compared among them using Student’s t-test based in both the standard error of the regressions and the standard error of the slopes^26^.

## Supplementary Information

Supplementary Information.

## Data Availability

All data is available in the main text or the supplementary materials (contact with Roque Serrano, e-mail: serrano@uji.es. Corresponding author).
