# Supplementary material for: Determination of very long-chain polyunsaturated fatty acids from 24 to 44 carbons in eye, brain and gonads of wild and cultured gilthead sea bream (Sparus aurata)
Source: Sci Rep. 2022 Jun 16;12:10112. doi: 10.1038/s41598-022-14361-0 (PMC9203556; doi:10.1038/s41598-022-14361-0)
Supplement: Supplementary file 1 — Supplementary Information. [file 41598_2022_14361_MOESM1_ESM.docx]

**SUPPLEMENTARY INFORMATION**

**Determination of very long-chain polyunsaturated fatty acids from 24 to 44 carbons in eye, brain and gonads of wild and cultured gilthead sea bream (*Sparus aurata*)**

Roque Serrano^*^, Juan C. Navarro, Carlos Sales, Tania Portolés, Óscar Monroig, Joaquin Beltran, Félix Hernández.

**
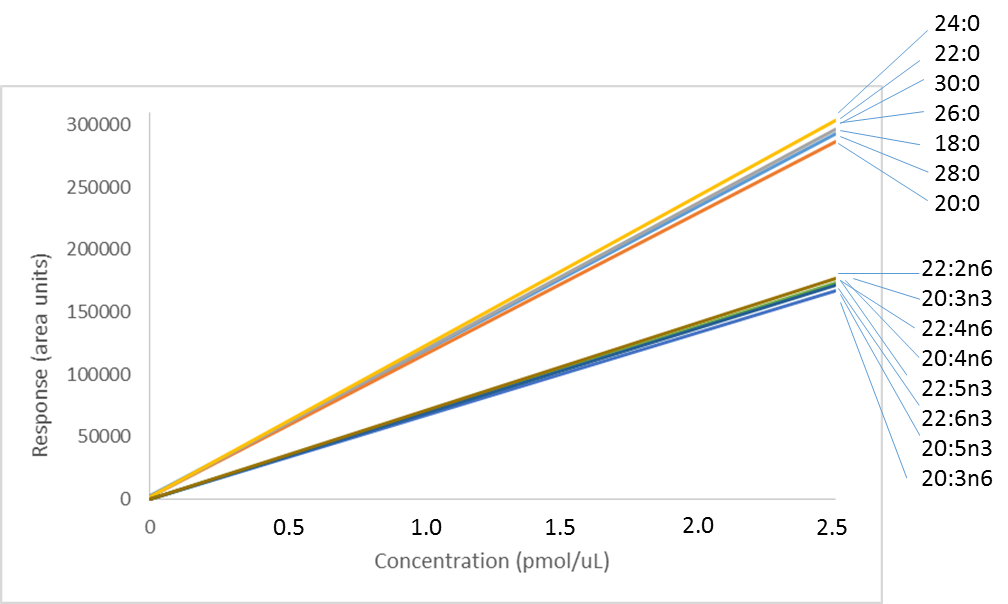
**

**Figure S1.** Calibration curves obtained by the method of the internal standard (27:0), injecting by triplicate 5 concentrations (0-2.5 pmol·µL^-1^) of each reference standard.

**
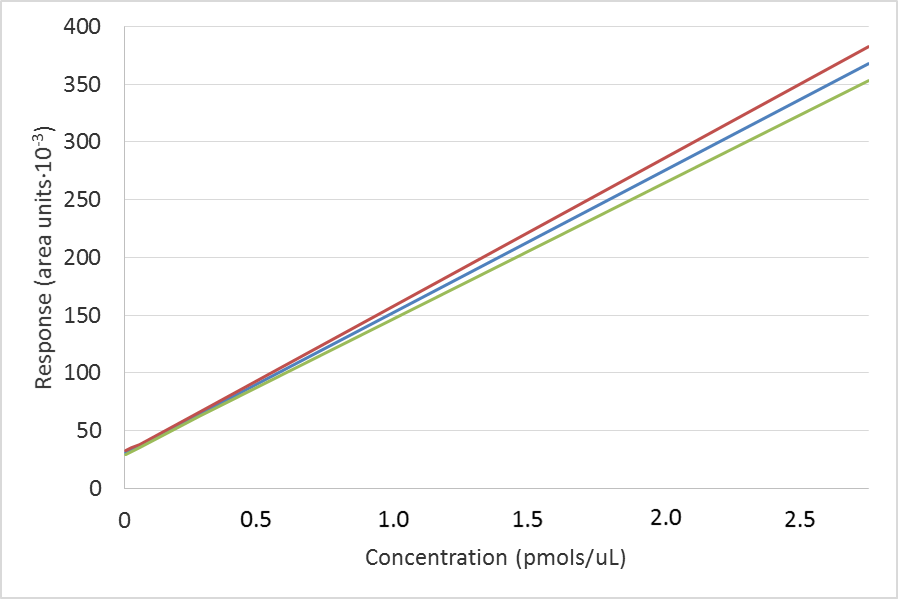
**

**Figure S2.** Confidence interval (95%, n=8) for the calibration curve obtained by calculating the arithmetic mean of responses of PUFA reference standards available (see Figure 1 SM).

**Table S1.** Mass spectrometry parameters used for the identification of targeted compounds.

**
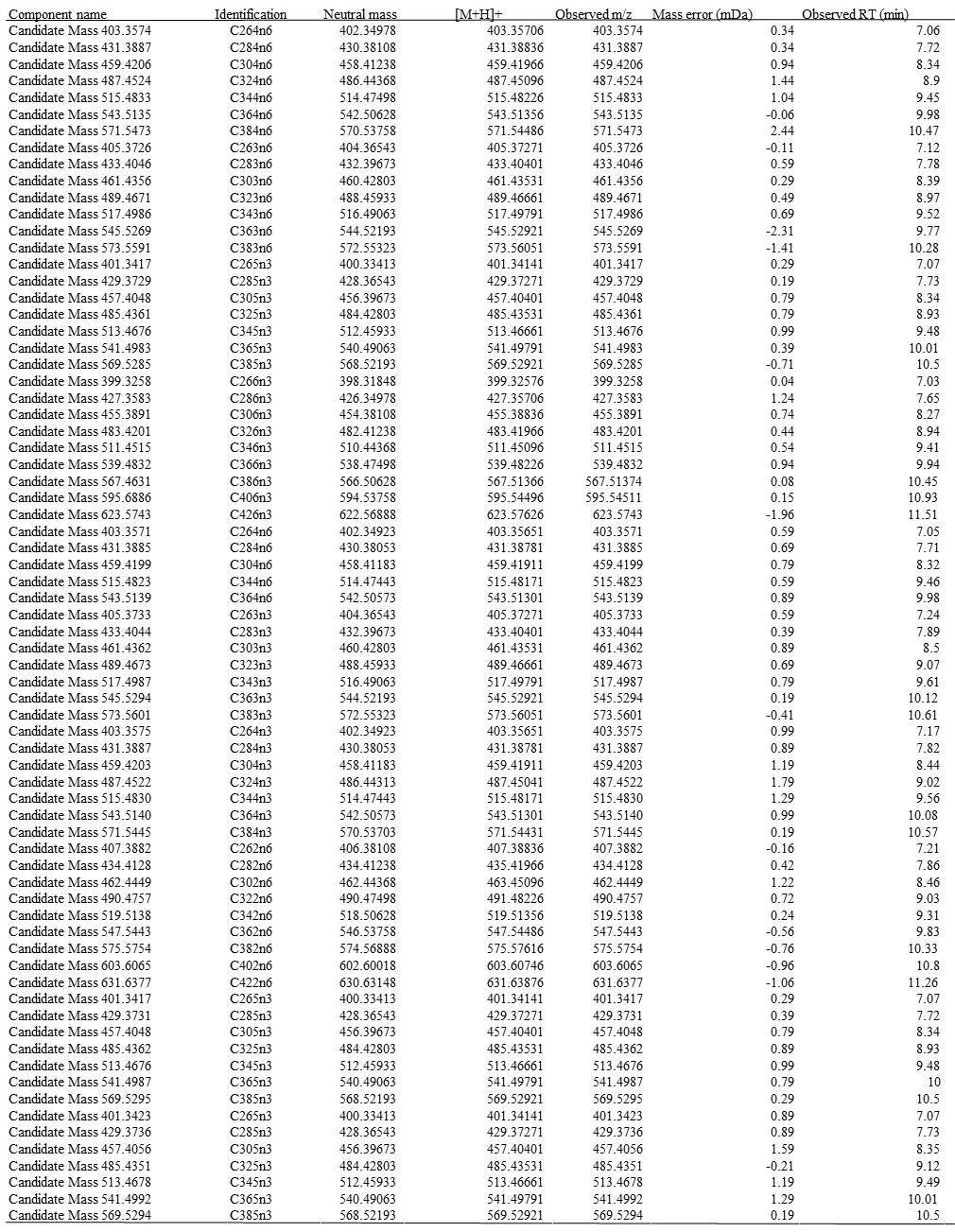
**

**Table S2 (A).** Quantification of VLC-PUFA contained in lipid classes from fish tissues (pmol/mg Lipid). Mean (n=6).

| **Compounds** |  | **24:5n6** | **24:6n3** | **24:4n6** | **26:5n6** | **26:6n3** | **26:4n6** | **28:6n3** | **28:4n6** | **28:5n3** | **30:6n3** | **30:4n6** |
| --- | --- | --- | --- | --- | --- | --- | --- | --- | --- | --- | --- | --- |
| *Wild* |  |  |  |  |  |  |  |  |  |  |  |  |
| Eye | SM | <LOQ | 0.0096 | 0.0042 | 0.0059 | <LOQ | 0.0051 | <LOQ | 0.0083 | <LOQ | 0.0068 | 0.0196 |
|  | PC | 0.0438 | 0.1434 | 0.0298 | 0.0319 | 0.0336 | 0.0137 | 0.0420 | 0.0138 | <LOQ | 0.2614 | 0.0396 |
|  | CER | 0.0040 | 0.0064 | <LOQ | <LOQ | <LOQ | <LOQ | <LOQ | <LOQ | <LOQ | 0.0051 | <LOQ |
| Gonads | SM | 0.0437 | 0.1741 | 0.0243 | 0.0126 | 0.0108 | 0.0087 | 0.0083 | 0.0071 | 0.0072 | 0.0126 | 0.0071 |
|  | PC | 0.2145 | 0.4752 | 0.2497 | 0.0574 | 0.0292 | 0.0523 | 0.0177 | 0.0222 | 0.0115 | 0.0225 | 0.0212 |
|  | CER | 0.0360 | 0.0303 | 0.0166 | 0.0098 | nd | 0.0085 | nd | nd | 0.0113 | nd | nd |
| Brain | SM | 0.0364 | 0.0834 | 0.0284 | 0.0144 | 0.0066 | 0.0041 | 0.0069 | 0.0056 | 0.0058 | 0.0075 | 0.0055 |
|  | PC | 0.2287 | 0.3722 | 0.0776 | 0.0658 | 0.0457 | 0.0209 | 0.0289 | 0.0104 | 0.0096 | 0.0222 | 0.0092 |
|  | CER | 0.0144 | 0.0224 | 0.0111 | 0.0094 | 0.0085 | 0.0058 | 0.0037 | 0.0025 | 0.0089 | nd | nd |
| *Farmed* |  |  |  |  |  |  |  |  |  |  |  |  |
| Eye | SM | nd | nd | nd | nd | nd | nd | nd | nd | nd | nd | nd |
|  | PC | <LOQ | 0.0333 | nd | nd | nd | nd | nd | nd | nd | 0.0518 | nd |
|  | CER | na | na | na | na | na | na | na | na | na | na | na |
| Gonads | SM | <LOQ | <LOQ | <LOQ | <LOQ | <LOQ | nd | nd | <LOQ | <LOQ | <LOQ | <LOQ |
|  | PC | 0.1865 | 0.5436 | nd | 0.0497 | 0.0144 | nd | <LOQ | nd | nd | nd | nd |
|  | CER | na | na | na | na | na | na | na | na | na | na | na |
| Brain | SM | 0.0050 | 0.0054 | 0.0050 | 0.0050 | 0.0050 | <LOQ | 0.0050 | <LOQ | 0.0054 | 0.0039 | <LOQ |
|  | PC | 0.3627 | 0.9371 | 0.0054 | 0.0119 | 0.0054 | 0.0054 | 0.0054 | 0.0054 | 0.0054 | 0.0054 | 0.0039 |
|  | CER | 0.0046 | 0.0050 | 0.0050 | <LOQ | <LOQ | nd | nd | nd | <LOQ | <LOQ | nd |

*SM: Sphingomyelin, PC: Phosphatidylcholine, CER: Cerebrosides*

**Table S2 (B).** Quantification of VLC-PUFA contained in lipid classes from fish tissues (pmol/mg Lipid). Mean (n=6).

| **Compounds** |  | **30:5n3** | **32:5n3** | **32:6n3** | **32:4n6** | **34:4n6** | **34:6n3** | **34:5n3** | **36:6n3** | **38:6n3** | **40:6n3** | **42:6n3** | **44:6n3** |
| --- | --- | --- | --- | --- | --- | --- | --- | --- | --- | --- | --- | --- | --- |
| *Wild* |  |  |  |  |  |  |  |  |  |  |  |  |  |
| Eye | SM | 0.0404 | 0.0159 | 0.0990 | 0.0062 | 0.0037 | 0.0099 | <LOQ | 0.0057 | <LOQ | 0.0058 | nd | nd |
|  | PC | 0.1140 | 0.3056 | 0.5000 | 0.0464 | 0.0062 | 0.1176 | 0.0115 | 0.0174 | 0.0085 | 0.0210 | <LOQ | nd |
|  | CER | 0.0053 | 0.0055 | 0.0712 | <LOQ | nd | <LOQ | <LOQ | <LOQ | <LOQ | <LOQ | nd | nd |
| Gonads | SM | 0.0089 | 0.0115 | 0.0327 | 0.0072 | 0.0070 | 0.0267 | 0.0080 | 0.0096 | 0.0070 | nd | nd | nd |
|  | PC | 0.0425 | 0.0283 | 0.0589 | 0.0191 | 0.0179 | 0.0367 | 0.0386 | 0.0260 | 0.0193 | 0.0156 | nd | nd |
|  | CER | nd | nd | 0.0118 | nd | nd | nd | nd | nd | nd | nd | nd | nd |
| Brain | SM | 0.0073 | 0.0064 | 0.0257 | <LOQ | <LOQ | 0.0084 | 0.0062 | 0.0133 | 0.0224 | 0.0935 | 0.0066 | nd |
|  | PC | 0.0160 | 0.0109 | 0.0333 | 0.0059 | 0.0092 | 0.0270 | 0.0133 | 0.0550 | 0.1349 | 0.4027 | 0.0200 | 0.0037 |
|  | CER | nd | nd | 0.0060 | nd | nd | 0.0044 | nd | <LOQ | 0.0025 | 0.0069 | nd | nd |
| *Farmed* |  |  |  |  |  |  |  |  |  |  |  |  |  |
| Eye | SM | nd | nd | 0.0154 | nd | nd | nd | nd | nd | nd | nd | nd | nd |
|  | PC | nd | 0.0206 | 0.5160 | nd | nd | 0.0186 | nd | nd | nd | nd | nd | nd |
|  | CER | na | na | na | na | na | na | na | na | na | na | na | na |
| Gonads | SM | <LOQ | <LOQ | <LOQ | nd | nd | <LOQ | <LOQ | <LOQ | <LOQ | nd | nd | nd |
|  | PC | nd | nd | nd | nd | nd | <LOQ | <LOQ | <LOQ | <LOQ | <LOQ | nd | nd |
|  | CER | na | na | na | na | na | na | na | na | na | na | na | na |
| Brain | SM | 0.0050 | 0.0039 | 0.0050 | nd | <LOQ | 0.0050 | 0.0050 | 0.0050 | 0.0050 | 0.0054 | <LOQ | nd |
|  | PC | 0.0054 | 0.0054 | 0.0054 | nd | <LOQ | 0.0054 | 0.0054 | 0.0054 | 0.0073 | 0.1525 | <LOQ | nd |
|  | CER | nd | nd | <LOQ | nd | nd | nd | nd | nd | nd | <LOQ | nd | nd |

*SM: Sphingomyelin, PC: Phosphatidylcholine, CER: Cerebrosides*

**Table S3 (A).** Quantification of VLC-PUFA contained in lipid classes from fish tissues (pmol/mg Lipid). Relative Standard Deviations (%)(n=6).

| **Compounds** |  | **24:5n6** | **24:6n3** | **24:4n6** | **26:5n6** | **26:6n3** | **26:4n6** | **28:6n3** | **28:4n6** | **28:5n3** | **30:6n3** | **30:4n6** |
| --- | --- | --- | --- | --- | --- | --- | --- | --- | --- | --- | --- | --- |
| *Wild* |  |  |  |  |  |  |  |  |  |  |  |  |
| Eye | SM |  | 26 | 17 | 16 |  | 17 |  | 21 |  | 14 | 26 |
|  | PC | 19 | 16 | 17 | 19 | 18 | 18 | 17 | 19 |  | 18 | 19 |
|  | CER | 11 | 11 |  |  |  |  |  |  |  | 16 |  |
| Gonads | SM | * | * | * | * | * | * | * | * | * | * | * |
|  | PC | 21 | 24 | 32 | 19 | 15 | 21 | 15 | 18 | 27 | 13 | 18 |
|  | CER | 21 | 18 | 29 | 35 |  | 40 |  |  | 27 |  |  |
| Brain | SM | 15 | 18 | 15 | 8 | 16 | 27 | 16 | 16 | 16 | 17 | 16 |
|  | PC | 16 | 15 | 15 | 10 | 13 | 7 | 6 | 6 | 6 | 5 | 6 |
|  | CER | 8 | 8 | 8 | 7 | 8 | 17 | 21 | 40 | 7 |  |  |
| *Farmed* |  |  |  |  |  |  |  |  |  |  |  |  |
| Eye | SM |  |  |  |  |  |  |  |  |  |  |  |
|  | PC |  | 23 |  |  |  |  |  |  |  | 23 |  |
|  | CER | na | na | na | na | na | na | na | na | na | na | na |
| Gonads | SM |  |  |  |  |  |  |  |  |  |  |  |
|  | PC | 35 | 35 |  | 35 | 35 |  | 35 |  |  |  |  |
|  | CER | na | na | na | na | na | na | na | na | na | na | na |
| Brain | SM | 15 | 11 | 15 | 15 | 15 |  | 15 |  | 11 | 23 |  |
|  | PC | 18 | 25 | 11 | 24 | 11 | 11 | 11 | 11 | 11 | 11 | 23 |
|  | CER | 15 | 15 | 15 |  |  |  |  |  |  |  |  |

*SM: Sphingomyelin, PC: Phosphatidylcholine, CER: Ceramides, *: n=1; na: not analyzed,* *blanks: nd or <LOQ values*

**Table S3 (B).** Quantification of VLC-PUFA contained in lipid classes from fish tissues (pmol/mg Lipid). Relative Standard Deviations (%)(n=6).

| **Compounds** |  | **30:5n3** | **32:5n3** | **32:6n3** | **32:4n6** | **34:4n6** | **34:6n3** | **34:5n3** | **36:6n3** | **38:6n3** | **40:6n3** | **42:6n3** | **44:6n3** |
| --- | --- | --- | --- | --- | --- | --- | --- | --- | --- | --- | --- | --- | --- |
| *Wild* |  |  |  |  |  |  |  |  |  |  |  |  |  |
| Eye | SM | 26 | 17 | 26 | 15 | 10 | 19 |  | 21 |  | 20 |  |  |
|  | PC | 21 | 20 | 4 | 18 | 15 | 19 | 17 | 18 | 16 | 18 |  |  |
|  | CER | 13 | 16 | 23 |  |  |  |  |  |  |  |  |  |
| Gonads | SM | * | * | * | * | * | * | * | * | * | * | * | * |
|  | PC | 19 | 16 | 11 | 18 | 20 | 14 | 20 | 18 | 20 | 22 |  |  |
|  | CER |  |  | 27 |  |  |  |  |  |  |  |  |  |
| Brain | SM | 15 | 17 | 26 |  |  | 16 | 16 | 15 | 15 | 7 | 15 |  |
|  | PC | 7 | 6 | 9 | 19 | 6 | 8 | 7 | 10 | 11 | 13 | 11 | 18 |
|  | CER |  |  | 16 |  |  | 23 |  |  | 40 | 24 |  |  |
| *Farmed* |  |  |  |  |  |  |  |  |  |  |  |  |  |
| Eye | SM |  |  | 37 |  |  |  |  |  |  |  |  |  |
|  | PC |  | 22 | 6 |  |  | 29 |  |  |  |  |  |  |
|  | CER | na | na | na | na | na | na | na | na | na | na | na | na |
| Gonads | SM |  |  |  |  |  |  |  |  |  |  |  |  |
|  | PC |  |  |  |  |  |  |  |  |  |  |  |  |
|  | CER | na | na | na | na | na | na | na | na | na | na | na | na |
| Brain | SM | 15 | 23 | 15 |  |  | 15 | 15 | 15 | 15 | 11 |  |  |
|  | PC | 11 | 11 | 11 |  |  | 11 | 11 | 11 | 15 | 27 |  |  |
|  | CER |  |  |  |  |  |  |  |  |  |  |  |  |

*SM: Sphingomyelin, PC: Phosphatidylcholine, CER: Ceramides, *: n=1, na: not analyzed, blanks: nd or <LOQ values*
